# Deep mutational scanning: A versatile tool in systematically mapping genotypes to phenotypes

**DOI:** 10.3389/fgene.2023.1087267

**Published:** 2023-01-12

**Authors:** Huijin Wei, Xianghua Li

**Affiliations:** ^1^ Zhejiang University—University of Edinburgh Institute, Zhejiang University, Haining, Zhejiang, China; ^2^ Deanery of Biomedical Sciences, University of Edinburgh, Edinburgh, United Kingdom; ^3^ The Second Affiliated Hospital of Zhejiang University, Hangzhou, Zhejiang, China; ^4^ Biomedical and Health Translational Centre of Zhejiang Province, Haining, Zhejiang, China

**Keywords:** deep mutational scanning, genotype-phenotype mapping, massively parallel mutagenesis, high-throughput analysis, systems biology, biotechnology

## Abstract

Unveiling how genetic variations lead to phenotypic variations is one of the key questions in evolutionary biology, genetics, and biomedical research. Deep mutational scanning (DMS) technology has allowed the mapping of tens of thousands of genetic variations to phenotypic variations efficiently and economically. Since its first systematic introduction about a decade ago, we have witnessed the use of deep mutational scanning in many research areas leading to scientific breakthroughs. Also, the methods in each step of deep mutational scanning have become much more versatile thanks to the oligo-synthesizing technology, high-throughput phenotyping methods and deep sequencing technology. However, each specific possible step of deep mutational scanning has its pros and cons, and some limitations still await further technological development. Here, we discuss recent scientific accomplishments achieved through the deep mutational scanning and describe widely used methods in each step of deep mutational scanning. We also compare these different methods and analyze their advantages and disadvantages, providing insight into how to design a deep mutational scanning study that best suits the aims of the readers’ projects.

## Introduction

**FIGURE 1:**
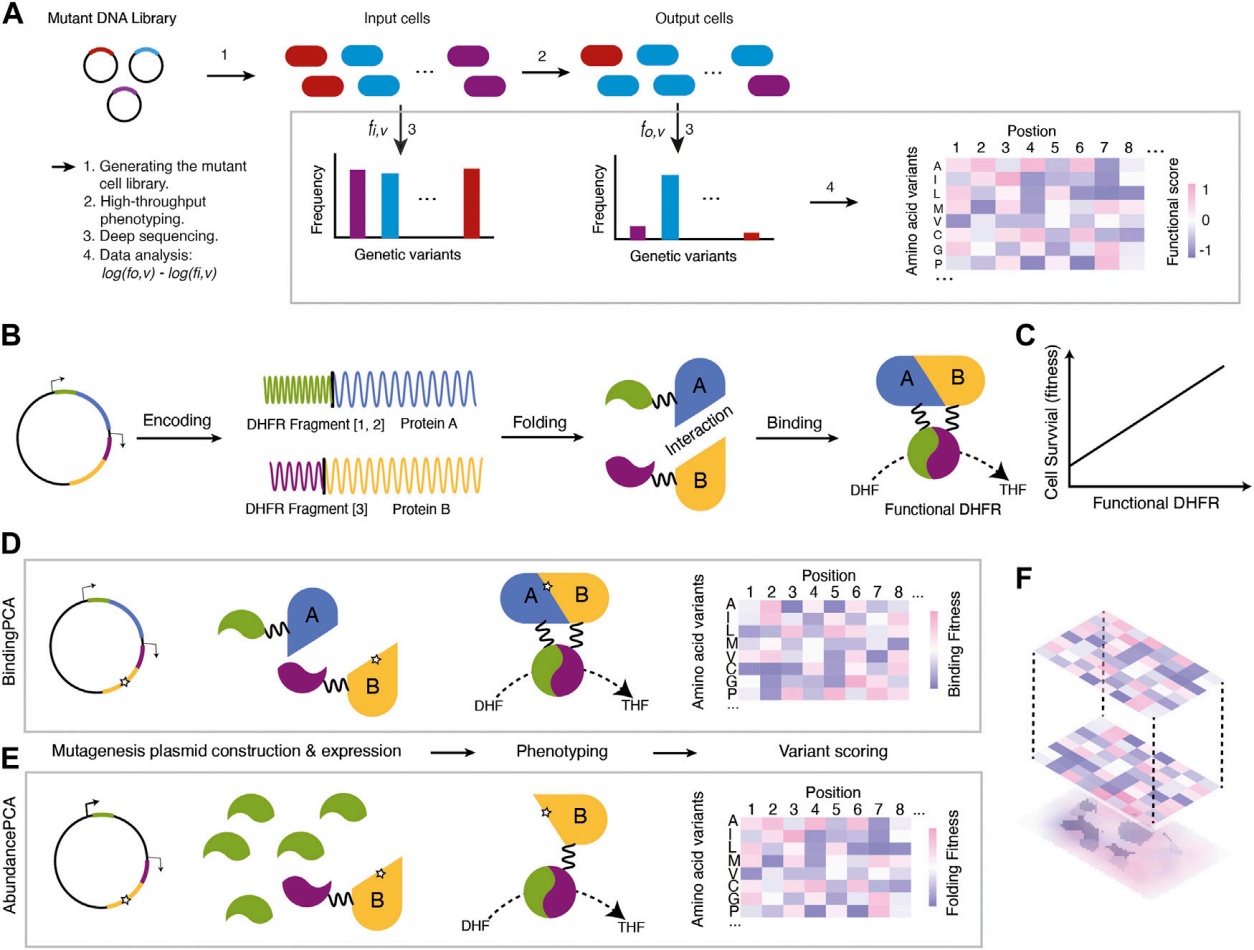
An overview of the DMS procedure **(A)** A mutant DNA library is transformed into cell types of interest to generate a mutant cell library. Then, the mutant cell library goes through high-throughput phenotyping where cells carrying functional variants are enriched (cells filled with blue) while those with detrimental variants are depleted (cells filled with red or purple). Genetic variants are extracted and sequenced to calculate the relative enrichment changes before and after selection. Finally, the enrichment scores are analysed as the functional scores of mutations **(B)** Protein-Fragment Complementation Assay (PCA) **(C)** The underlying assumption is that the concentrations of functional DHFR are linearly related to cell survival (fitness) **(D)** BindingPCA captures mutational effects on both stability and protein-protein interactions without distinguishing them **(E)** AbundancePCA captures mutational effects on stability **(F)** ddPCA combines BindingPCA and AbundancePCA and enables the inference of the bbphysical effects of mutations by quantifying and comparing phenotypic effects.

Since Mendel’s experiments with peas (Mendel, 1865) laid the foundation of modern genetics about 150 years ago, our ability to read, write, and rewrite genetic information has grown prominently. In comparison, our ability to understand genetic information—i.e., mapping genetic variations to phenotypic variations—is very limited. For instance, the effects of the vast majority of human genetic variations are unknown (Riesselman et al., 2018; Frazer et al., 2021; Lappalainen and MacArthur, 2021). In light of this challenge, deep mutational scanning (DMS) was developed to systematically quantify the effects of genetic variations on a large scale, with high efficiency and relatively low cost (Fowler et al., 2011; Hietpas et al., 2012). DMS, also known as massively parallel mutagenesis (Fowler et al., 2011; Fowler and Fields, 2014), involves making a comprehensive mutant library followed by high-throughput phenotyping and deep-sequencing of the mutant libraries before and after selection (Figure 1A).

DMS has been widely used in many biological systems, allowing breakthroughs in biological and biomedical research since its first introduction (Fowler et al., 2010; Fowler and Fields, 2014). For example, many human disease-related genetic variants with unknown significance have been classified as either benign or detrimental systematically (Majithia et al., 2016; Findlay et al., 2018; Matreyek et al., 2018; Mighell et al., 2018; Bridgford et al., 2020; Mighell et al., 2020; Hanna et al., 2021; Seuma et al., 2021). Genetic interaction patterns and the underlying biophysical mechanisms have been revealed for both between genes (Diss and Lehner, 2018; Lite et al., 2020; Faure et al., 2022) and within the same gene (Olson et al., 2014; Li et al., 2016; Puchta et al., 2016; Sarkisyan et al., 2016; Baeza-Centurion et al., 2019; Yoo et al., 2020; Faure et al., 2022). Also, using the positional genetic interaction scores generated from DMS experiments, protein structures can be accurately predicted (Rollins et al., 2019; Schmiedel and Lehner, 2019). The release of the DMS data on SARS-Cov2 spike protein RBD within a year of the SARS-Cov2 outbreak (Starr et al., 2020) demonstrates that DMS is a powerful technique to address pressing questions in a relatively short period. The data accurately captured some SARS-Cov2 mutations that became prevalent in the later stage of the pandemic (Starr et al., 2020; Starr et al., 2022). Furthermore, DMS data on immune-escape mutants of various SARS-Cov2 variants (Greaney et al., 2021a; Greaney et al., 2021b; Javanmardi et al., 2022) guides better vaccine design.

A typical DMS experiment involves three steps: 1) generating a genetic mutant library; 2) performing a high-throughput phenotyping assay; 3) and deep sequencing and data analysis. Several good reviews on designing DMS experiments were published (Fowler and Fields, 2014; Shin and Cho, 2015; Starita and Fields, 2015; Matuszewski et al., 2016; Cao et al., 2022) in the early days of DMS. However, many more technical options became available in DMS thanks to the fast-developing technology in gene synthesis, sequencing technologies and high-throughput phenotyping methods since the reviews. The recent reviews (Weile and Roth, 2018; Kemble et al., 2019; Kinney and McCandlish, 2019; Narayanan and Procko, 2021; Hanning et al., 2022) in light of the DMS boom mostly focus on specific biological insights—for example, how the technique enabled breakthroughs in human genetics (Weile and Roth, 2018), on transcriptional factors (TF) and cis-regulatory elements (CRE) (Kinney and McCandlish, 2019), on viral protein and receptors (Narayanan and Procko, 2021) or therapeutic antibody engineering (Hanning et al., 2022). Kemble et al. gave a comprehensive overview of genotype-phenotype mapping (Kemble et al., 2019) enabled by DMS technology. While the DMS strategy is straightforward, each step of the technique can be tricky and complicated to generate clean and meaningful data, as it involves various synthetic biology and massive parallel assays. In addition, genetic variants from DMS experiments are of low complexity but are of a big amount that needs special attention for statistical analysis. We notice a lack of such up-to-date reviews on the insights in technical aspects.

In this review, we give an up-to-date overview of the DMS experiment (Figure 1A) with a specific focus on recently developed techniques in mutation library generation, high-throughput methods, and data analysis. Our motivation is to guide the readers on selecting the most appropriate techniques for a DMS project aim. Finally, we will discuss the ongoing efforts and challenges in improving DMS accuracy and scope.

## Generating a genetic mutant library

A genetic mutant library is often first synthesized as a pool of oligos and amplified as a library of linear gene blocks. Then the amplified dsDNA is ligated to the expression vector backbones to substitute the wild-type region of the gene to be mutated. The ligation mix is introduced to the cloning cell lines to be amplified and extracted as a plasmid mutant library, which will be introduced to the destination cells (i.e., *via* transformation) for the next step—high throughput phenotyping assay. While most of the steps mentioned above follow regular molecular cloning procedures, mutagenesis in the very first step is not trivial and requires careful design. In this section, we will discuss the most widely used methods in designing and creating mutant libraries so that the readers can determine the optimal method to suit their research needs.

### Error-prone PCR

Error-prone PCR is relatively cheap and easy to perform. It uses low-fidelity DNA polymerases to incorporate mistakes during the DNA amplification, and mutation rates can be modified by PCR conditions like different concentrations of manganese chloride and dNTP (Lin-Goerke et al., 1997; Shafikhani et al., 1997). The technique has been widely used in making random mutations for directed evolution experiments (Giver et al., 1998; Moore et al., 2000) and recently for DMS studies (Sarkisyan et al., 2016; Seuma et al., 2021; Faure et al., 2022). However, mutations generated *via* error-prone PCR are not completely random due to mutation biases of polymerases. For example, Taq polymerase-based mutation rates from A/T are much higher than from C/G (Shafikhani et al., 1997; Wan et al., 1998). Nowadays, error-prone PCR is made easier using commercial kits with mixes of engineered polymerases (Vanhercke et al., 2005), generating reduced biases. However, judging from the DMS data (Faure et al., 2022), mutation biases are only partially removable even with commercial kits. To be noted, error-prone PCR is suitable for generating comprehensive nucleotide-level mutations but not for all possible single amino acid substitutions for each codon. To achieve all possible 19 amino acid substitutions per codon, two consecutive nucleotides of a codon must often be mutated simultaneously. But such a mutation rate will likely hit two or more codons simultaneously, creating a mutation library mixed with single amino acid substitutions and multiple amino acid substitutions.

### PCR with oligonucleotides containing mutations

Another commonly used method is a DMS library with a pool of oligos containing different mutations. Compared to the error-prone PCR, it is more costly but can generate a customized library with fewer biases. Oligonucleotides containing random mutations can be synthesized as a pool of doped oligos (Matteucci and Heyneker, 1983; Araya et al., 2012; Li et al., 2016; Puchta et al., 2016; Li et al., 2019; Wu et al., 2022) or oligos containing NNN triplets (sometimes NNS or NNK) (Hietpas et al., 2012; McLaughlin et al., 2012; Stiffler et al., 2015; Starr et al., 2017; Diss and Lehner, 2018; Hartman et al., 2018; Ahler et al., 2019; Starr et al., 2020; Park et al., 2022), where N represents any of the four nucleotide bases, S for G/C and K for G/T) targeting each codon. This strategy, combined with oligo pool synthesis technology like DropSynth (Plesa et al., 2018), allows construction of user-defined, scalable, and low-cost mutant libraries with comprehensive nucleotide or amino acid substitutions.

These oligos can be designed as doped oligos with each position incorporating a defined percentage of mutations (Starita and Fields, 2015) during the oligo synthesis. The pool of the long mutant oligos (up to 300 nt) can be used as DNA templates. These long oligos need to contain flanking wild-type sequences for primer binding, so they can be amplified and replace the wild-type sequences. On the other hand, short oligos with user-defined mutations or NNN triplets serve as primers. Mutations are introduced to the gene in a manner that is similar to site-directed mutagenesis. The oligos containing NNN triplets are more suited to create mutant libraries covering all possible single amino acid substitutions, while this doped oligo method also targets nucleotide-level mutations as error-prone PCR does. The disadvantage of using oligos with NNN triplets is that it often requires at least two consecutive PCR reactions to generate double amino acid substitutions.

Another popular primer-based method is the nicking mutagenesis (Wrenbeck et al., 2016; Faure et al., 2022), which is developed from a method called Pfunkle (Firnberg and Ostermeier, 2012). Both methods use the circular dsDNA as the template and incorporate mutations using a mix of phosphorylated primers. To remove excessive wild-type template, thymidine in the template is replaced with uracil and degraded after the mutagenesis using the uracil DNA glycosylase and exonuclease III (Exo III) (Firnberg and Ostermeier, 2012). For the same purpose, nicking mutagenesis uses a pair of endonucleases (NtBbvCl and NbBbvCl) that nick one strand of the template dsDNA at a time.

Firstly, a 5’ phosphorylated mutant oligo pool as primers is applied to the NtBbvCI-treated ssDNA template to generate the second-strand DNA with mutations. Then, a second phosphorylated primer without mutations will synthesize the complementary strand for each mutated genetic variant, using the PCR-derived strand with mutations as templates. While other primer-based mutagenesis methods require a pair of primers per mutant, both Pfunkle and nicking mutagenesis requires only one primer per mutant, greatly reducing the cost of oligo synthesis. But to perform Pfunkle or nicking mutagenesis, one needs to ensure high-quality circular DNA and careful design of the primer libraries with a freshly phosphorylated state. Nevertheless, nicking mutagenesis has been rising in popularity for achieving codon-level saturation mutagenesis recently.

### Generating a library with mutations at the endogenous genetic loci

For DMS studies aimed at endogenous genetic loci, CRISPR-based technologies (Doudna and Charpentier, 2014; Rees and Liu, 2018) are used. The mutant library can be designed as a sgRNA library targeting intended genetic loci (Wang et al., 2014; Hart et al., 2015; Sadhu et al., 2018; Hanna et al., 2021) or as a donor DNA mutant library for homology-directed repair (HDR) template (Findlay et al., 2014; Sharon et al., 2018; Choudhury et al., 2020; Shen et al., 2022). The donor DNA mutant library can be generated using the methods mentioned above and combined into the backbone flanked by the recombination arms and necessary components. Simultaneous use of two gRNAs also enables multiplexed mutagenesis (Campa et al., 2019). Yet, compared to the ectopic expression of a mutation library, there are much fewer DMS studies performed at the endogenous loci due to additional technical limitations—including sgRNA-dependent uneven editing efficiencies (Wang et al., 2014; Bassalo et al., 2018; Choudhury et al., 2020), low HDR efficiency (Findlay et al., 2014), and high incidences of undetected off-target mutations and editing biases (Cui and Bikard, 2016; Zerbini et al., 2017). The challenges are even more prominent when the mammalian cell genome is the target of saturation mutagenesis (Rees and Liu, 2018).

To sum up, different methods of generating mutation libraries have their own pros and cons (Table 1). The choice of the method should be determined primarily by the purpose. For instance, should the experiment target nucleotide or codon level, single or combinations of mutations? How extensive the mutant library should be, and should the mutations be ectopically expressed or integrated into the genome?

**TABLE 1:** Mutant library construction.

—	*Targeted mutations*	*Number of mutations*	*Pros*	*Cons*
*Error-prone PCR*	Nucleotide (nt) level	A distribution of single and multiple changes, by modifying the PCR conditions	Economical; Easy to perform	Mutation bias
*Doped oligo*	Nucleotide (nt) level	A distribution of single and multiple changes, designed as an error rate per position (i.e., 1.2% error rate/position)	Economical; Customized mutation distribution	Oligo size is limited by the coupling efficiency. The longer the oligos are, the lower the oligo pool qualities are. It is limited up to 300 nt
*NNN (NNE or NNS) oligos*	Amino acid (AA) level	All possible single AA per codon	Comprehensive protein residue substitution effects; Can be designed as primers or PCR templates	Two or more rounds of PCR required to achieve multi-codon mutants
*Gene blocks for Homology- Directed Repair (HDR)*	Nucleotide or amino acid (AA) level	A distribution of single and multiple changes	Endogenous expression of the mutant variants	Delivery and HDR efficiency limit the library size
*sg-RNA library*	Nucleotide (nt) level	Single mutants but with possible off-target mutations	Endogenous expression of the mutant variants	Off-target issues

## High-throughput phenotyping

After obtaining the mutant plasmid library or gene blocks *via* various molecular cloning steps, including amplification, ligation, *etc.*, the library is delivered (*via* transformation, transfection, or transduction) to the cell types of interest for high-throughput quantification of the phenotypes coupled by the deep sequencing. These phenotyping assays are usually designed to enrich functional genetic variants while depleting the detrimental variants in a bulk experiment (Fowler and Fields, 2014; Olson et al., 2014; Bandyopadhyay et al., 2020) or *via* reporter-based cell sorting (Starr et al., 2017; Matreyek et al., 2018; Li et al., 2019; Park et al., 2022).

Measured phenotypes can be divided into two main categories: 1) Fitness based on the reproduction rate of cells (Li et al., 2016; Puchta et al., 2016; Domingo et al., 2018) or 2) measurement of the molecular function (abundance, binding, or activity) (Araya et al., 2012; Olson et al., 2014; Sarkisyan et al., 2016; Matreyek et al., 2018; Li et al., 2019; Tack et al., 2021; Faure et al., 2022). In this section, we will describe and compare techniques used in these two categories and another recently developed method that can decompose molecular functions *via* the fitness-based assay.

### Fitness assays

Fitness competition is the most straightforward and economical approach for a high-throughput functional selection. Its logic is that if the gene product is required for cell survival or reproduction, cells carrying functional genetic variants will enrich. In contrast, detrimental variants will deplete over time in a culture medium. As a result, frequency changes of genetic variants can be calculated as fitness scores (Domingo et al., 2018). This strategy does not require special equipment, making it easy to conduct. However, the obtained fitness scores may not necessarily be linearly related to the molecular mechanisms of the mutations, making it complicated to acquire mechanistic insight into the mutational effects on the molecular level (Stein et al., 2019). Besides, marginally detrimental effects on molecular functions may be masked due to the non-linear relationship between fitness and molecular function (Soskine and Tawfik, 2010; Stiffler et al., 2015). It also needs to be noted that mutational effects often alter in different environments (Stiffler et al., 2015; Domingo et al., 2018; Chen et al., 2022).

Therefore, it is essential to select an optimal condition that either reflects the physiological situation best (Starita et al., 2015; Braun et al., 2018; Cantor et al., 2018; Hartman et al., 2018; Staller et al., 2018; Ahler et al., 2019; Matreyek et al., 2020; Mighell et al., 2020) to unveil disease-causing mutations or evolutionary paths of mutations. On the other hand, to easily infer biophysical effects, the fitness assay conditions should be selected to be linearly related to the molecular function (Domingo et al., 2018; Li et al., 2019; Leander et al., 2020; Starr et al., 2020; Faure et al., 2022).

### Functional assays

Using the protein stability or binding affinity as a phenotype (Araya et al., 2012; Olson et al., 2014; Starr et al., 2020; Faure et al., 2022) is another widely used method to evaluate mutational effects for a protein-coding gene. This approach can capture essential biophysical effects of mutations and give more mechanistic insights into mutations.

Stability assays often involve tagging the target protein to a reporter, like the green fluorescent protein (GFP) as an indicator of the protein stability (Li et al., 2019; Leander et al., 2020; Matreyek et al., 2020; Park et al., 2022). Cells can be sorted based on the fluorescence levels into several bins, followed by deep sequencing of each sorted subpopulation (Peterman and Levine, 2016; Matreyek et al., 2018). Then, each mutant’s mean fluorescence level is calculated based on the frequencies of each genetic variant in each sorted bin.

*In vitro* display methods, such as phage display (Araya et al., 2012), yeast display (Klesmith et al., 2017; Starr et al., 2017; Cao et al., 2022), and mRNA display (Olson et al., 2014), detect frequency changes of genetic variants based on the binding affinity of the protein to its ligands. Although the experimental results from such an approach reveal the functional effects of mutations, it does not immediately indicate whether mutations affect the function by changing the protein stability or binding affinity, which is termed biophysical ambiguity hereafter. Nevertheless, it is crucial to resolve the biophysical ambiguity of mutations if we want to predict the combined effects of mutations (Otwinowski et al., 2018; Li and Lehner, 2020) accurately. To overcome this, approaches like combining the binding affinity-based functional assay and the stability-based assay (Starr et al., 2020), or predicting mutants’ biophysical effects by analyzing how mutations combine based on a single assay (Otwinowski et al., 2018) have been shown.

Performing two sets of different experiments (Starr et al., 2020) are often troublesome while predicting folding and binding energy changes based on the protein structures (Capriotti et al., 2005; Schymkowitz et al., 2005; Zhang et al., 2020) is not as accurate as experiment results. Recently, a method called ddPCA (Faure et al., 2022) that uses a relatively simple experimental approach to solve the biophysical ambiguity has been developed, which we will discuss in the following part.

### ddPCA: Untangling biophysical parameters with the fitness assays

The method called Double Deep Protein-Fragment Complementation Assay (ddPCA) (Faure et al., 2022) is based on the protein-fragment complementation (PCA) assay (Tarassov et al., 2008). In ddPCA, the expression ratios of dihydrofolate reductase (DHFR) fragments are tweaked into two sets so that one assay can detect mutational effects on the stability of the protein (AbundancePCA) while the other detects both stability and protein-protein interactions (BindingPCA) (Figures 1B–F).

BindingPCA uses the traditional PCA method in which two interacting partners are each tagged with interacting partners are each tagged with DHFR[1,2] and DHFR[3] fragments (Figure 1D). Mutations that affect the binding affinity to the ligand and/or the protein stability will reduce the functional DHFR concentration inside the cells and therefore minimize cell survival (fitness) (Figures 1B, C). AbundancePCA, on the other hand, only has one protein-coding gene tagged to one fragment of DHFR (DHFR[3]) while overexpressing the other fragment DHFR[1,2]. This allows the cellular fitness to be solely determined by the limiting concentration of the protein tagged with DHFR fragment (i.e., DHFR [1,2], which reflects the protein stability (Figure 1E). The combination of the BindingPCA and the AbundancePCA serves to determine biophysical effects and resolve biophysical ambiguities (Figure 1F). Compared to other experimental approaches, ddPCA is a much simpler approach to unveil stability and binding affinity of mutations because both AbundancePCA and BindingPCA use the same fitness selection system. ddPCA has been applied to several allosteric proteins and resolves the ‘biophysical ambiguities’, as well as pinpointing allosteric sites systematically (Faure et al., 2022; Weng et al., 2022).

Besides the methods mentioned above, enzyme kinetics can be measured in a dynamic system using microfluidics technology. For instance, the High-Throughput Microfluidic Enzyme Kinetics (HT-MEK) in a DMS experiment allows the systematic investigation of enzymes in an automatically valved microfluidics expression system (Markin et al., 2021).

## Deep sequencing and data analysis

Deep-sequencing of the genetic variants for both the input (before phenotyping) and the output (after phenotyping) follows the high-throughput phenotyping. Samples from a DMS experiment are special in that there are up to tens of thousands of genetic variants. Yet, they are with a low frequency of mutations at each position (sometimes as low as 0.1%) in an overall very homogenous sequence. Considering that genotype-phenotype mapping depends on frequencies of each genetic variant that are often only one or two hamming distances away from each other, choosing a high-throughput sequencing platform with high accuracy is especially important for a DMS study.

### Sequencing platforms

The most widely used platform in DMS studies has been the Illumina HiSeq platforms for their relatively lower error rates compared to the third-generation sequencing platforms (PacBio or Nanopore sequencing) and the higher cost-effectiveness (cost/base pair) compared to Illumina MiSeq (Shendure et al., 2017; Pfeiffer et al., 2018). However, the HiSeq platforms have a sequencing read length limitation to 300 nt. This makes the identification of long-range epistatic interactions challenging if the mutated region exceeds the length limit. To overcome this, barcoding genetic variants can be applied, first to associate genetic variants with barcodes using Miseq or PacBio sequencing (Puchta et al., 2016; Starr et al., 2020; Wu et al., 2022) and then to perform deep sequencing of the barcodes using HiSeq.

Recently, UMI-based Nanopore sequencing (Zurek et al., 2020) and new circular consensus sequencing (CCS) method using PacBio (Wenger et al., 2019) were developed to increase the accuracies of long-read sequencing platforms to >99.5% (Zurek et al., 2020; Karst et al., 2021). This suggests that Nanopore or PacBio may completely substitute HiSeq for DMS studies in the future.

Experiment design and library preparation for sequencing are utterly important to obtain high-quality data, regardless of the sequencing platforms used. One should always 1) start with a sufficient number of molecules per variant in each mutant library; 2) have multiple independent biological replicates; and 3) avoid experimental bottlenecks, over-sequencing or under-sequencing. Especially, it is essential not to over-sequence as that would impactfully hinder accurate prediction of the variant frequencies (Faure et al., 2020). Read-depths should not be more than that of total expected molecule numbers before generating sequencing libraries but also need to be sufficiently bigger than the expected unique counts of genetic variants.

### Data analysis

The phenotype of each genetic variant is often quantified as the normalized relative enrichment scores from the aggregated count data (i.e., after *versus* before selection) compared to that of the wild type, as shown in Eq. 1 below.

$$E_{v\;}=\log2\frac{F_{v,output}}{F_{v,input}}-\log2\frac{F_{wt,output}}{F_{wt,input}}$$ (1)

*F*_*v,output*_ and *F*_*v,input*_ are the frequencies of a given variant *v* after selection (*output*) and before selection (*input*) respectively, and *E*_*v*_ is the normalized enrichment score of the variant to the wild type. When the phenotype is based on the reporter fluorescence intensity and cell sorting (Starr et al., 2020), functional scores as the mean fluorescence signals are estimated based on the variant counts in each sorted bin and the bin fluorescence parameters (Peterman and Levine, 2016).

To estimate mutants’ phenotypes from the sequencing data accurately and to obtain enough statistical power, correct error detection and propagation are essential. However, it is not a simple task as there are many sources of errors in the typical DMS dataset, including sequencing error, Poisson error, errors from the replicates, and stochastic error (Rubin et al., 2017). Enrich2 (Rubin et al., 2017) and a more recent software DiMSum (Faure et al., 2020) are two good statistical frameworks developed for DMS sequencing data to help users reliably quantify the data and perform error estimation. Enrich2 and DiMSum are both based on the Poisson-based sequence count distribution, and they both integrate the empirical variance into account to estimate the errors. However, the way they handle the empirical variance is different. For instance, Enrich2 takes the mix-effects from the empirical variance, while DiMSum introduced replicate-specific additive and multiplicative modifier terms from empirical variance. Thus, the error estimated from the two models differs (Faure et al., 2020). The only available and direct comparisons between the two statistical software are from Faure and others who developed DiMSum. Based on the 12 datasets examined, Enrich2 and DiMSum performed similarly well on the datasets with little overdispersion, but Enrich2 underestimates errors on the dataset with a lot of overdispersion (Faure et al., 2020). Still, DiMSum is not as widely used as Enrich2 in DMS data analysis, likely because it is still a relatively newly developed pipeline. Either error models from Enrich2 or DiMSum cannot capture systematic errors arising from the experiments, which need to be identified and judged by the researchers using diagnostic plots. After this step, one could select only reliable data based on the error thresholds for further analysis.

Relative mutational effects are often presented in a 2D map, with each x- and *y*-axis representing mutation position and substitution respectively, and the phenotype in a gradient of filled color as a heatmap (Figure 1). With such a descriptive figure giving an overview of the data, one can quickly judge which positions are more sensitive to mutations and whether certain types of substitutes are more acceptable than others. There are also tools developed to visualize both published and own DMS data. MaveVis, developed as part of the MaveDB (Esposito et al., 2019), allows users to generate heatmaps integrating the protein structural information for each position. It is available for both web-based interfaces and as an R package. Another web-based tool called dms-view (Hilton et al., 2020) can provide a quick exploration of the DMS data to look for specific mutations per site and in the context of protein 3D structure in an interactive manner. Compared to MaveVis, the advantage of dms-view is the integration of the protein 3D structure and logo generation based on mutational effects. However, local users cannot use the software as it is only web-based.

To obtain mechanistic insights into genotype-phenotype maps, machine-learning algorithms (Wang et al., 2014; Hart et al., 2015; Majithia et al., 2016; Klesmith et al., 2017; Rocklin et al., 2017; Weile et al., 2017; Gray et al., 2018; Staller et al., 2018; Song et al., 2021; Faure et al., 2022; Hsu et al., 2022; Leander et al., 2022) and deep learning algorithms (Sarkisyan et al., 2016; Gelman et al., 2021; Faure et al., 2022; Tareen et al., 2022; Vaishnav et al., 2022) are frequently used. Especially, a recently developed python package called MAVE-NN (Tareen et al., 2022) can unveil the one-dimensional latent phenotypes (i.e., a hidden type of biophysical parameter values) that are non-linearly linking genotypes to phenotypes, based on the neural-network algorithm. MAVE-NN has its limitations. For example, it cannot analyse DMS data with only single mutations or mutations affecting more than one type of expected biophysical parameters to reveal the latent phenotypes.

## Discussion

Recent years have witnessed a boom in DMS applied to various coding and non-coding genes from many organisms, including viruses, bacteria, yeast, and mammalian cells. In this review, we presented an overview of DMS that combines synthetic biology, high-throughput phenotyping methods, and deep sequencing technology. We also showed the main steps in the DMS technique and compared different choices of designing mutation libraries, phenotyping assays, and sequencing platforms. Finally, by comparing different techniques, we gave brief guidance on selecting the most appropriate strategy according to different scientific questions and experimental models.

A high-quality DMS dataset not only provides important information on genotype-phenotype mapping for biomedical research, but also guides other research fields including structural biology, biophysics, and protein engineering. For instance, the very comprehensive single and double-mutant GB1 DMS dataset (Olson et al., 2014) enabled accurate prediction of the protein 3D structure (Schmiedel and Lehner, 2019) and biophysical effects of each mutation without doing the painstaking experiments (Gelman et al., 2021; Tareen et al., 2022). Also, the technology provides mechanistic insights into understanding and predicting mutational effects (Hsu et al., 2022), contributing to protein engineering and structure prediction (Rollins et al., 2019; Schmiedel and Lehner, 2019), biomedicine (Braun et al., 2018; Baeza-Centurion et al., 2019; Bridgford et al., 2020; Livesey and Marsh, 2020; Frazer et al., 2021) and evolution (Aakre et al., 2015; Li et al., 2016; Puchta et al., 2016; Starr et al., 2017; Domingo et al., 2018; Starr et al., 2020; Park et al., 2022; Starr et al., 2022). In light of accumulating DMS datasets and the challenge of reproducibility and source-data compilation, several pioneering labs in the field of massive parallel assays made an open-source platform called MaveDB (Esposito et al., 2019) available for DMS experiment data. By now (December 2022), more than a hundred DMS datasets have been listed in MaveDB that are available for download and analyse. An alliance called Atlas of Variant Effects (https://www.varianteffect.org) is also formed to maximise collaboration, benefits, and the influence of mutational scanning.

Still, there are limitations in DMS technology. Firstly, each DMS experiment could handle up to tens of thousands of mutations but not more. One of the limiting factors in scaling mutation libraries is transformation (transfection or transduction) efficiencies, as one does not want to generate a bottleneck by randomly sampling genetic variants that go into the destination cells. The number of successfully transformed (transfected or transduced) cells should be sufficiently higher than the library sizes to minimize the loss of some genetic variants during the transformation step. Secondly, performing DMS at the endogenous genomic loci of cells is still a big challenge. Nevertheless, endogenous DMS will become available soon with improved precision in genome editing technologies and transformation/transfection efficiencies. While DMS has been applied to various organisms, including humans, viruses, bacteria and yeast, interestingly, there is no DMS research on plant genes, even though mapping genotypes to phenotypes on the plant is both important and challenging (Voichek and Weigel, 2020; Deng et al., 2021). The reason could be the technical challenges in developing a high throughput phenotyping assay with the designed mutation pools.

To sum up, our ability to interpret genotypes is still lacking due to the complication of the genotype-phenotype maps (Domingo et al., 2019; Kinney and McCandlish, 2019). While sequencing technology is becoming more advanced and economical, ‘reading’ genetic codes has become the routine of many labs. We believe that DMS will become a laboratory routine in the near future together with further development in synthetic biology and sequencing technologies.
